# Ebselen enhances insulin sensitivity and decreases oxidative stress by inhibiting SHIP2 and protects from inflammation in diabetic mice

**DOI:** 10.7150/ijbs.66314

**Published:** 2022-02-14

**Authors:** Zydrune Polianskyte-Prause, Tuomas A. Tolvanen, Sonja Lindfors, Kanta Kon, Laura C. Hautala, Hong Wang, Tsutomu Wada, Hiroshi Tsuneki, Toshiyasu Sasaoka, Sanna Lehtonen

**Affiliations:** 1 Research Program for Clinical and Molecular Metabolism, University of Helsinki, Helsinki, Finland.; 2 Department of Pathology, University of Helsinki, Helsinki, Finland.; 3 Department of Clinical Pharmacology, University of Toyama, Toyama, Japan.

**Keywords:** insulin resistance, inflammation, lipid phosphatase, oxidative stress, type 2 diabetes

## Abstract

Ebselen, a multifunctional organoselenium compound, has been recognized as a potential treatment for diabetes-related disorders. However, the underlying mechanisms whereby ebselen regulates metabolic pathways remain elusive. We discovered that ebselen inhibits lipid phosphatase SHIP2 (Src homology 2 domain-containing inositol-5-phosphatase 2), an emerging drug target to ameliorate insulin resistance in diabetes. We found that ebselen directly binds to and inhibits the catalytic activity of the recombinant SHIP2 phosphatase domain and SHIP2 in cultured cells, the skeletal muscle and liver of the diabetic db/db mice, and the liver of the SHIP2 overexpressing (SHIP2-Tg) mice. Ebselen increased insulin-induced Akt phosphorylation in cultured myotubes, enhanced insulin sensitivity and protected liver tissue from lipid peroxidation and inflammation in the db/db mice, and improved glucose tolerance more efficiently than metformin in the SHIP2-Tg mice. SHIP2 overexpression abrogated the ability of ebselen to induce glucose uptake and reduce ROS production in myotubes and blunted the effect of ebselen to inhibit SHIP2 in the skeletal muscle of the SHIP2-Tg mice. Our data reveal ebselen as a potent SHIP2 inhibitor and demonstrate that the ability of ebselen to ameliorate insulin resistance and act as an antioxidant is at least in part mediated by the reduction of SHIP2 activity.

## Introduction

Oxidative stress, an imbalance between oxidative and antioxidative systems of cells and tissues, results from overproduction of reactive oxygen species (ROS). ROS act as intracellular signaling messengers and are important in moderate amounts for a number of physiological processes. However, excessive levels of ROS can lead to cellular damage of lipids, proteins and DNA, and impair cellular functions including energy metabolism and cell signaling 1, 2.

Increased oxidative stress has been implicated in the development of insulin resistance, impaired insulin secretion and altered glucose tolerance that can ultimately contribute to the onset and progression of diabetes and its associated complications, such as cardiovascular disease and diabetic nephropathy 1, 2. Furthermore, oxidative stress-induced chronic inflammation is associated with the development of type 2 diabetes (T2D) 3.

Lipid phosphatase SHIP2 serves a target to treat insulin resistance, as it suppresses phosphatidylinositol 3-kinase (PI3K)-mediated insulin signaling pathway by hydrolyzing phosphatidylinositol (3,4,5)-trisphosphate (PI(3,4,5)P3) to phosphatidylinositol (3,4)-bisphosphate (PI(3,4)P2) 4, 5. In human, polymorphisms in *INPPL1*, the gene encoding SHIP2, are associated with the development of hypertension, the metabolic syndrome and T2D 6. SHIP2 is upregulated in adipose tissue, skeletal muscle and kidney glomeruli of diabetic rodents 7, 8. Transgenic mice overexpressing SHIP2 and mice overexpressing SHIP2 in liver have impaired glucose metabolism and insulin sensitivity 9, 10. SHIP2 knockout mice are resistant to high fat-diet induced obesity 11 and liver-specific inactivation of SHIP2 in diabetic mice *via* overexpression of dominant-negative mutant of SHIP2 ameliorates glucose metabolism and insulin resistance 9, 12. In various cell culture models, overexpression of a dominant-negative mutant of SHIP2 enhances insulin signaling 13, ameliorates insulin resistance induced by pro-inflammatory cytokine TNFα 14 and reduces ROS production induced by palmitate or high glucose 15, 16. These data support the attractiveness of inhibiting SHIP2 activity for the treatment of insulin resistance induced by elevated oxidative stress and inflammation in T2D.

To discover new SHIP2 inhibitors, we performed *in silico* structure-based virtual screening of small molecule chemical libraries and identified ebselen, in addition to the well-known antidiabetic drug metformin 17, as a novel SHIP2 inhibitor. Ebselen, a synthetic multifunctional organoselenium compound, prevents ROS-induced cellular damage by mimicking the glutathione peroxidase activity 18 and exhibits antioxidant and anti-inflammatory properties in different experimental models 19. Ebselen has been in Phase II-III clinical trials for the treatment of various disorders such as cerebral ischemia, bipolar disorder and noise-induced hearing loss (see clinicaltrials.gov website for more information). In diabetes-related disorders, ebselen improves glucose-stimulated insulin secretion in murine islets 20, shows beneficial effects on β-cell mass and function in a diabetic rat model 21 and attenuates hyperglycemia induced by either organo-phosphate diazinon in rats 22 or ischemia/reperfusion injury in gerbils 23. However, the exact molecular mechanism by which ebselen regulates metabolic pathways and reduces hyperglycemia remains unclear.

In this study, we investigate whether ebselen enhances insulin sensitivity and protects from oxidative stress and inflammation by inhibiting SHIP2 activity. We also compare the ability of ebselen and metformin to improve glucose tolerance. We demonstrate that ebselen directly binds to and reduces the activity of the phosphatase domain of SHIP2, providing a potential molecular mechanism by which ebselen ameliorates insulin resistance and protects against oxidative stress and inflammation.

## Materials and methods

### Cell culture and biochemical assays

Rat L6 myoblasts (ATCC, Manassas, VA, USA) were maintained in high glucose (4.5 g/L) DMEM (MilliporeSigma, Burlington, MA, USA) and hepatoma cells (Fao cells, Public Health England, Salisbury, UK) in F-12K Nutrient Mixture media (Gibco, ThermoFisher Scientific, Waltham, MA, USA) according to the provider's instructions. Generation of L6 myoblasts stably expressing hemagglutinin-GLUT4-green fluorescent protein (HA-GLUT4-GFP, referred to as L6-GLUT4) has been previously described 17. Lentiviral infection was used to overexpress SHIP2 in L6-GLUT4 myotubes on day 10-12 of differentiation for 72 h as previously described 8. Lipofectamine 3000 (Invitrogen, ThermoFisher Scientific) was used to transiently overexpress human SHIP2 cDNA in pCGN vector or empty pCGN 8 in L6 myotubes on day 3-4 of differentiation for 48 h according to the provider's instructions. JetPRIME transfection reagent (Polyplus-transfection Inc., Illkirch, France) was used to transiently silence SHIP2 gene with *INPPL1* siRNAs (ON-TARGETplus Rat *Inppl1* siRNA SMART pool #L-080026-02-0010, Dharmacon, Horizon Discovery, Cambridge, UK) in L6 myoblasts for 72 h according to the provider's instructions. Non-targeting siRNA pool (ON-TARGETplus Non-targeting pool #D-001810-10-05, Dharmacon) was used as a control. Differentiation of L6 myoblasts started one day after transient transfection. Myotubes, hepatoma cells and liver tissue were lysed and immunoblotting performed as previously described 24. Skeletal muscle tissue was lysed with Precellys ceramic beads (Bertin Corp, Rockville, MD, USA) using 1% *n*-Dodecyl-β-D-maltoside in PBS (MilliporeSigma) supplemented with 1 mM phenylmethylsulfonyl fluoride and phosphatase/protease inhibitors as previously described 24. The antibodies used are listed in Supplementary Table S1. Glucose uptake assay was performed as previously described 24. Cytokine concentrations were measured from liver tissue lysates with Quansys Q-Plex ELISA-based cytokine assay (Quansys Bioscience, Logan, UT, USA) according to the provider's instructions and normalized to protein concentration. ROS production was assessed as in 25 with minor modifications. Cells were washed with phenol red-free DMEM (Gibco) and incubated with 50 µM 2',7'-dichlorofluorescein diacetate (DCFH-DA) fluorescent probe and 2 µM Hoechst 33342 (MilliporeSigma) at 37 °C for 30 min. After washing once with phenol red-free DMEM, the fluorescence intensity was measured at 485/535 nm using a Hidex Sence microplate reader (Hidex, Turku, Finland).

### Production of recombinant SHIP2 and SHIP1 phosphatase domains, and SHIP2 immunoprecipitation

Recombinant His-tagged human SHIP2 and SHIP1 phosphatase domains were produced as previously described 26. SHIP2 was immunoprecipitated as in 17 from lysates of myotubes and hepatoma cells treated with ebselen (MilliporeSigma) at indicated concentrations and times or from lysates of tissues of mice treated with ebselen (Tocris Bioscience, Bristol, UK or Cayman Chemicals, MI, USA) for 12 days or 10 weeks and mice treated with metformin (MilliporeSigma) for 12 days.

### Enzyme activity measurements

The catalytic activities of the recombinant SHIP2 and SHIP1 phosphatase domains, and SHIP2 immunoprecipitated from cell and tissue lysates were determined by malachite green phosphate assay using PI(3,4,5)P3 diC8 at the final concentration of 100 µM (Echelon Biosciences, Salt lake City, UT, USA) as previously described 17.

### Cellular thermal shift assay (CETSA)

Cells were lysed and CETSA performed as previously described 27. Briefly, myotubes were harvested, washed with PBS and cell pellet resuspended in cold PBS supplemented with 6 mM MgCl_2_ and 1xComplete^TM^-EDTA free proteinase inhibitor cocktail (Roche, Basel, Switzerland). Cell suspensions were freeze-thawed three times using liquid nitrogen and the soluble fraction was separated by centrifugation at 20'000 g for 20 min at +4°C. The cell lysates were treated with 50 µM ebselen or corresponding volume of DMSO as a control and incubated for 15 min at room temperature. The lysates were divided into 12 aliquots and heated individually at different temperatures for 3 min in PCR machine (Veriti thermal cycle, Applied Biosystems) followed by cooling for 5 min at room temperature. Subsequently, the heated lysates were centrifuged at 20'000 g for 20 min at +4°C and supernatants were separated by SDS-PAGE followed by Western blot analysis.

### Metabolic assays in db/db and SHIP2-Tg mice

Male C57BL/Ks-db/db (BKS.Cg-m+/+Lepr/OlaHsd) mice (ENVIGO, Venray, Netherlands) and SHIP2-Tg mice 10 were maintained according to the principles of laboratory animal care, and the experiments were approved by the National Animal Experiment Board. 8-10 mice per group were used in the studies to account for unexpected animal loss during experiment. Randomly assigned 2-3 months old SHIP2-Tg mice received ebselen, resuspended in 0.5% sodium carboxymethyl cellulose (MilliporeSigma) at a dose of 10 mg/kg, by gavage twice a day for 12 days with a final dose administered by gavage 4 h prior to sacrifice. Metformin (MilliporeSigma) was administered to 4-5 months old SHIP2-Tg mice in drinking water (250 mg/kg/day) for 12 days with a final dose administered by gavage 4 h prior to sacrifice. SHIP2-Tg control mice of similar ages received 0.5% sodium carboxymethyl cellulose or plain drinking water, respectively. After 1 week of acclimation, db/db mice were randomly assigned to experimental groups based on body weight and fasting blood glucose levels. db/db mice were fed from 6 weeks of age for 10 weeks with Purina 5001 (Research Diets Inc., NJ, USA) or Purina 5001 containing ebselen 75 mg/kg (reformulated by Research Diets Inc.). This provided an average dosing of 10 mg ebselen/kg/day. Insulin tolerance test (after 10 weeks of ebselen treatment of the db/db mice) using human insulin (0.75 U/kg) (Actrapid, Novo Nordisk, Denmark) and glucose (2 g/kg) tolerance test (after 11 days of ebselen or metformin treatment of the SHIP2-Tg mice) were performed after 6 h fasting. Blood glucose concentrations were measured with Bayer's Elite (db/db mice) or FreeStyle Kissei Glucometer (SHIP2-Tg mice). At the end of the experiments, mice were euthanized, and blood was collected by cardiac puncture. Freshly dissected skeletal muscle and liver tissues were snap-frozen in liquid nitrogen, embedded in Tissue-Tek^®^ OCT compound (Sakura Finetek, Netherlands), or fixed in 10% formalin followed by embedding in paraffin. Serum lipid parameters of the db/db mice were analyzed at the Biochemical Analysis Core for Experimental Research (University of Helsinki). Quantitative RT-PCR was performed as previously described 28. The primer pairs are listed in Supplementary Table S2.

### Immunohistochemical analysis

Paraffin sections were deparaffinized and antigens retrieved with 10 mM Tris-EDTA buffer (pH 9.0) or with Dako Flex high pH buffer (Dako, Agilent, Santa Clara, CA, USA). Sections were blocked with 10% goat serum and 0.1% BSA in Tris-buffered saline or CAS blocking buffer (Life technologies, Carlsbad, CA, USA) for 30 min and stained with anti-4-HNE or F4/80, respectively, using BrightVision polyHRP-anti-Rabbit IgG kit (ImmunoLogic, Amsterdam, Netherlands) and 3,3'-diaminobenzidine (DAB) (Dako) followed by counterstaining with hematoxylin. Sections were stained with anti-fibronectin using EnVison polyHRP-anti-rabbit IgG kit (Dako). Sections were stained with anti-3-NT and anti-8-OHdG using mouse on mouse kit (Abcam, Cambrindge, UK) according to the provider's instructions. Hepatic lipid accumulation was analyzed by Oil Red O staining of frozen liver sections. Slides were digitally scanned using 3DHISTECH Pannoramic 250 FLASH II scanner (Genome Biology Unit, University of Helsinki) and quantified with HistoQuant module (3DHISTECH Ltd., Budapest, Hungary).

### Statistical Analyses

Data are shown as mean ± SD or mean ± SEM. Statistical analyses were performed using two-tailed Student's *t*-test for comparison of two groups and one-way or two-ways ANOVA with Tukey's post-hoc test for multiple comparisons. Western blot quantification data for the CETSA melting curves were normalized and fitted to the Boltzmann Sigmoid equation within GraphPad Prism (San Diego, CA, USA). *P*-values of < 0.05 were considered as statistically significant. Image analysis of the tissue sections was performed blindly by one investigator. Otherwise, randomization and blinding were not carried out. Due to welfare issues one control db/db and one control SHIP2-Tg mice were excluded from the study.

## Results

### Ebselen inhibits SHIP2 in myotubes and hepatoma cells

**Figure 1:**
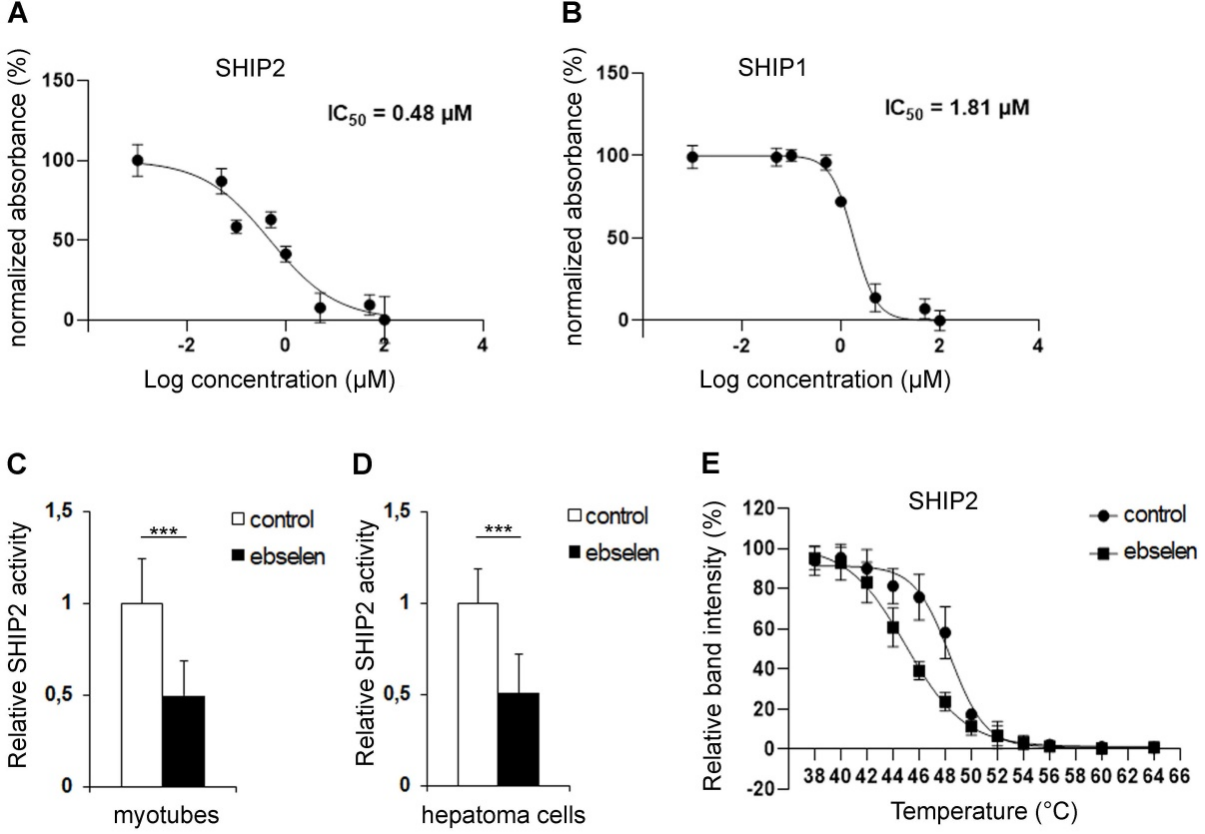
** Ebselen binds to and reduces the catalytic activity of SHIP2. (A and B)** Dose-dependent inhibition of the activities of the recombinant human SHIP2 (A) and SHIP1 (B) phosphatase domains with ebselen. The catalytic activity of SHIP2 and SHIP1 was measured by malachite green phosphate assay at the High Throughput Biomedicine Unit (University of Helsinki, Helsinki, Finland). Signal output is converted to percent of normalized absorbance. Data are presented as means ± SD of two-three technical replicates in each concentration point.** (C and D)** Ebselen reduces the activity of SHIP2 in L6 myotubes (C) and hepatoma cells (D). Cells were treated with 10 µM ebselen and the corresponding volume of DMSO as a control for 20-24 h. SHIP2 was immunoprecipitated from cell lysates and its catalytic activity measured by malachite green phosphate assay. **(E)** Binding of ebselen induces a shift in the melting temperature of SHIP2. CETSA melting curves for SHIP2 in myotybe lysates treated with 50 µM ebselen or corresponding volume of DMSO as a control. Data are presented as means ± SD of three-four independent experiments. Student's *t* test. ***p ˂ 0.001.

Ebselen was identified, together with metformin, in an *in silico* virtual screening of small molecule libraries in search for novel SHIP2 inhibitors 17. To confirm that ebselen inhibits the catalytic activity of SHIP2, we carried out malachite green phosphate assays. We found that ebselen directly binds to and reduces the catalytic activity of the recombinant SHIP2 phosphatase domain in a concentration-dependent manner with an IC_50_ value of 0.48 µM (Figure 1A). Ebselen inhibited also the recombinant SHIP1 phosphatase domain in a concentration-dependent manner with an IC_50_ value of 1.81 µM (Figure 1B). Enrichment of SHIP2 by immunoprecipitation from cultured myotubes and hepatoma cells followed by the measurement of the phosphatase activity in the precipitate revealed that 10 µM ebselen reduces SHIP2 activity by approximately 50% in both cell types (Figure 1C, D) without affecting SHIP2 expression level (Supplementary Figure S1A-D). To further confirm the ability of ebselen to bind to SHIP2, we carried out cellular thermal shift assay (CETSA) 29, which validates drug-target engagement in cells. Ebselen shifted the melting temperature of SHIP2 by -3.4°C in myotube lysates, indicating that ebselen directly binds to SHIP2 causing its destabilization (Figure 1E and Supplementary Figure S1E).

### Ebselen, but not metformin, improves glucose tolerance of the SHIP2-Tg mice

**Figure 2:**
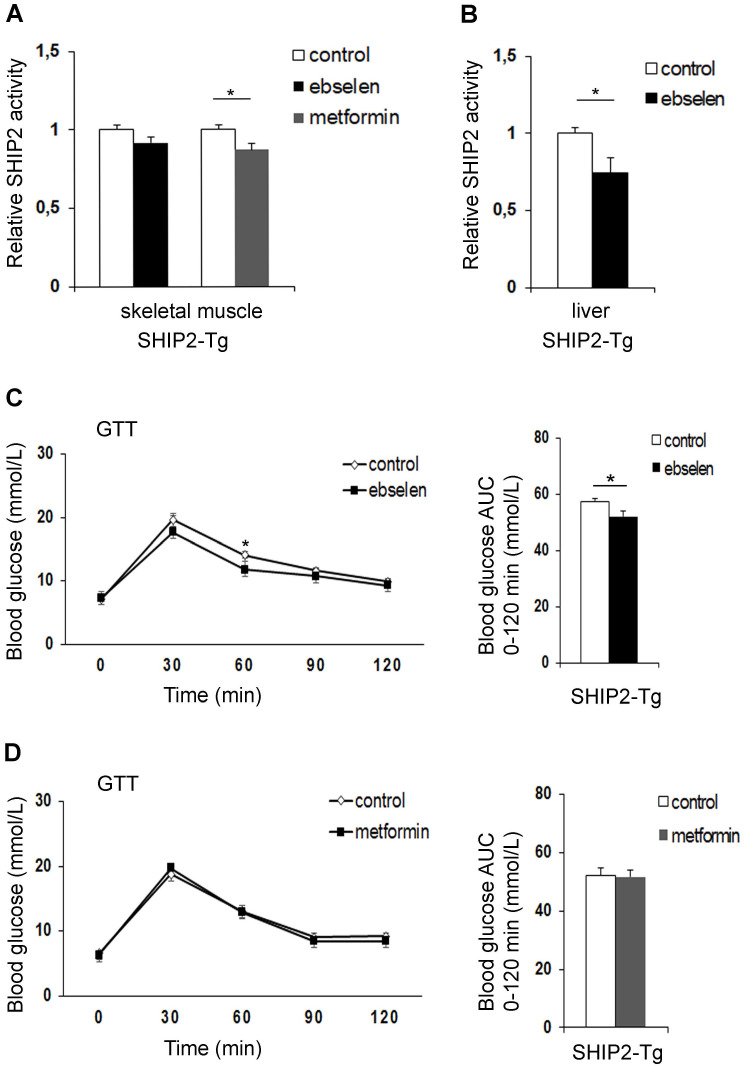
** Ebselen reduces the catalytic activity of SHIP2 in the liver of the SHIP2-Tg mice and improves glucose metabolism *in vivo*. (A)** Ebselen has no effect and metformin reduces the activity of SHIP2 in the skeletal muscle of the SHIP2-Tg mice. **(B)** Ebselen reduces the activity of SHIP2 in the liver of the SHIP2-Tg mice. SHIP2 was immunoprecipitated from tissue lysates and its catalytic activity measured by malachite green phosphate assay. **(C and D)** Ebselen (C) improves glucose tolerance whereas metformin (D) has no effect on glucose tolerance in the SHIP2-Tg mice. Glucose tolerance test (GTT) was performed and blood glucose measured at the indicated times after glucose administration, and the area under the curve (AUC) was calculated. Data are presented as means ± SEM control n=8-9; ebselen n=9-10; metformin n=8. Student's *t* test. *p ˂ 0.05.

To compare the effectiveness of ebselen versus metformin *in vivo*, we treated transgenic mice overexpressing SHIP2 under β-actin promoter (SHIP2-Tg mice) with ebselen or metformin for 12 days. These mice show overexpression of SHIP2 in different tissues, including skeletal muscle and liver 10. Ebselen had no effect and metformin inhibited the catalytic activity of SHIP2 by 12% in the skeletal muscle (Figure 2A). In the liver, ebselen inhibited the catalytic activity of SHIP2 by 25% (Figure 2B). For metformin-treated mice, we did not measure SHIP2 activity in the liver as our earlier data revealed that metformin does not inhibit SHIP2 in the liver or hepatoma cells 17. Ebselen or metformin treatment had no effect on SHIP2 expression level in the tissues of the SHIP2-Tg mice (Supplementary Figure S2A-D)*.* SHIP2-Tg mice show impaired glucose metabolism in normal chow diet 10. We observed that short-term treatment of the SHIP2-Tg mice with ebselen or metformin had no effect on body weight and fasting blood glucose compared to the nontreated SHIP2-Tg mice (Supplementary Table S3). However, glucose tolerance test revealed that ebselen reduces fasting blood glucose at 60 min after glucose administration and lowers the glucose area under the curve when compared to the nontreated SHIP2-Tg mice (Figure 2C). The blood glucose concentrations and the glucose area under the curve were unchanged in the metformin-treated SHIP2-Tg mice compared to the nontreated SHIP2-Tg mice (Figure 2D). These data show that ebselen improves glucose tolerance of the SHIP2-Tg mice more efficiently than metformin.

### Ebselen inhibits SHIP2 in the muscle and liver of the db/db mice

**Figure 3:**
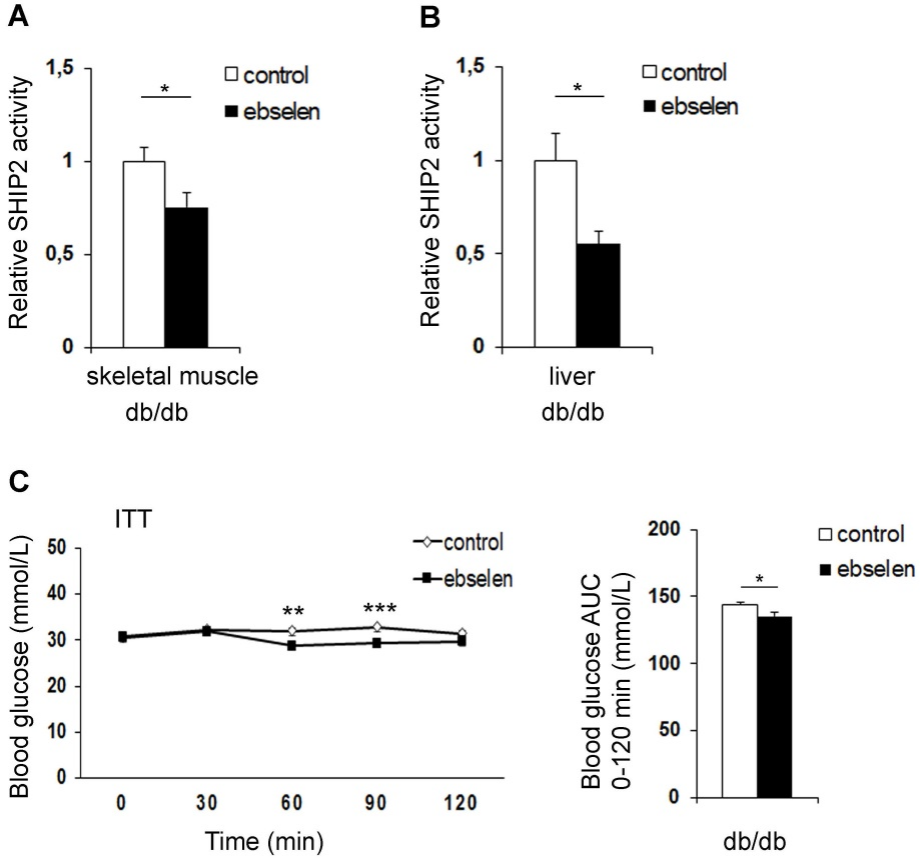
** Ebselen reduces the catalytic activity of SHIP2 in the skeletal muscle and liver of the db/db mice and improves insulin sensitivity *in vivo*. (A and B)** Ebselen reduces the activity of SHIP2 in the skeletal muscle (A) and liver (B) of the db/db mice. SHIP2 was immunoprecipitated from tissue lysates and its catalytic activity measured by malachite green phosphate assay. **(C)** Ebselen improves insulin sensitivity of the db/db mice. Insulin tolerance test (ITT) was performed and blood glucose measured at the indicated times after insulin injection, and the area under the curve (AUC) was calculated. Data are presented as means ± SEM control n=8-9; ebselen n=10. Student's *t* test. *p ˂ 0.05, **p ˂ 0.01, ***p ˂ 0.001.

Next, to determine whether ebselen inhibits SHIP2 in an *in vivo* model of T2D, we used diabetic db/db mice. The db/db mice were treated with ebselen for 10 weeks, yielding a dose of 13.4 mg/kg/day in the beginning and 8.2 mg/kg/day in the end of the experiment due to weight gain of the mice (Supplementary Table S4). Ebselen treatment decreased the enzymatic activity of SHIP2 by 25% in the skeletal muscle (Figure 3A) and by 48% in the liver (Figure 3B) without affecting SHIP2 expression level in either tissue (Supplementary Figure S3A-D).

### Ebselen ameliorates insulin resistance and increases the expression of antioxidant SOD1 in the muscle of the db/db mice

Treatment of the db/db mice with ebselen had no effect on body weight, even though food consumption was decreased at 8-9 weeks of treatment (Supplementary Table S4). Fasting blood glucose concentrations were similar between the ebselen-treated and nontreated db/db mice (Supplementary Table S5). Insulin tolerance test revealed that ebselen reduces fasting blood glucose at 60-90 min after insulin administration and lowers the glucose area under the curve when compared to the nontreated db/db mice (Figure 3C). Ebselen treatment decreased serum total cholesterol and tended to decrease low-density lipoprotein (LDL) (p=0.08) levels, while triglycerides, high-density lipoprotein (HDL) and non-esterified fatty acid (NEFA) levels were unchanged compared to the nontreated db/db mice (Supplementary Table S5). Ebselen did not alter the expression level of catalase, but increased the expression level of another antioxidant, SOD1 (Supplementary Figure S3A, B) in the skeletal muscle. Collectively, these data indicate that ebselen enhances insulin sensitivity in the db/db mice and increases the expression of SOD1 in the muscle.

### Ebselen increases the phosphorylation of Akt and enhances glucose uptake into myotubes by inhibiting SHIP2 activity

**Figure 4:**
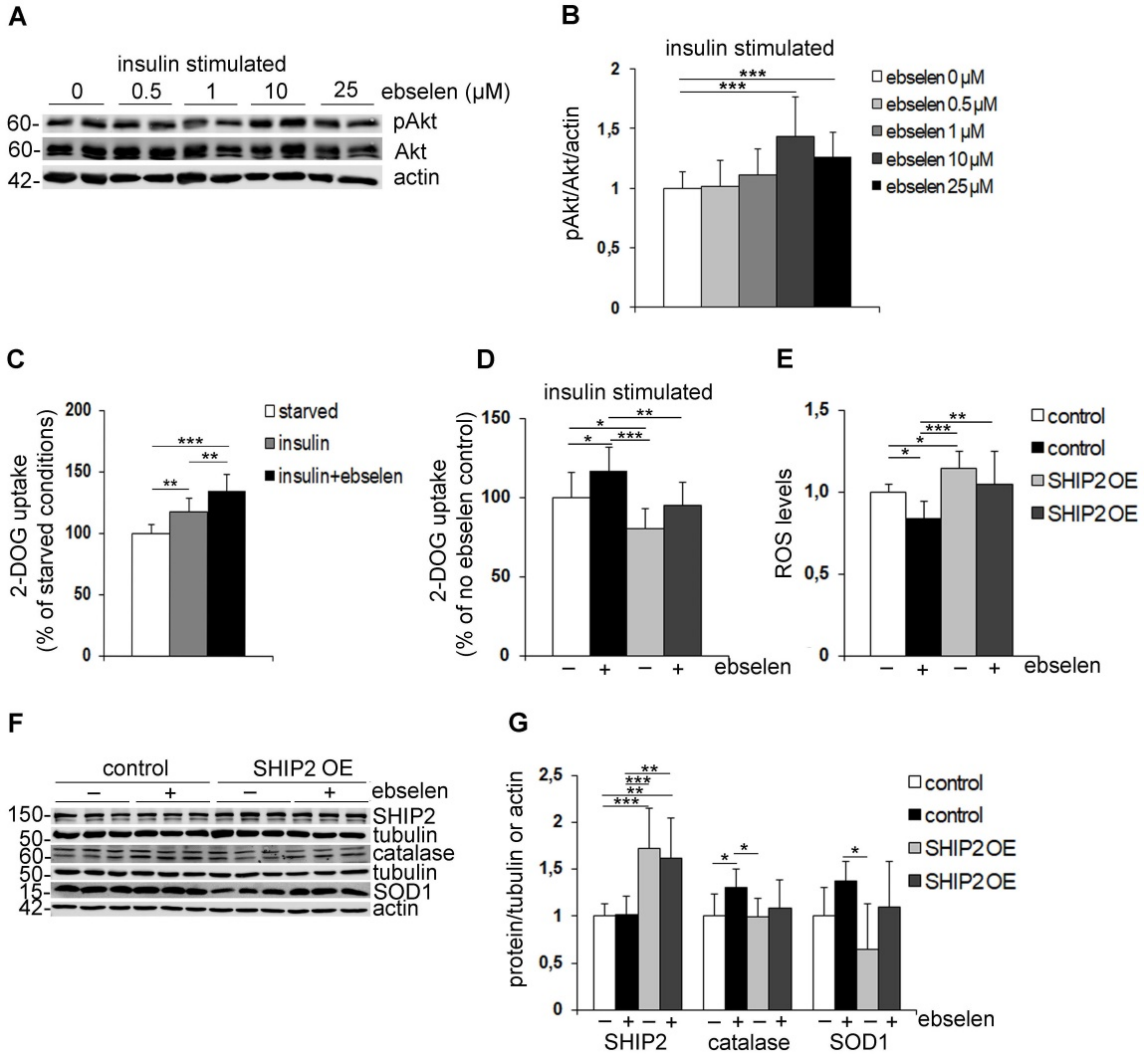
** Ebselen enhances insulin signaling, induces glucose uptake, increases the expression levels of antioxidant enzymes and reduces ROS production by inhibiting SHIP2. (A)** Representative immunoblots showing that ebselen dose-dependently increases insulin-induced Akt phosphorylation (pAkt) in L6 myotubes. Serum-starved myotubes (20 h) were pretreated for 15 min with ebselen at indicated concentrations or corresponding volume of DMSO as a control followed by stimulation with 10 nM insulin for an additional 15 min. Cell lysates were subjected to immunoblot analysis with anti-Akt, anti-pAkt (Ser 437) and anti-actin IgGs. **(B)** Quantification of pAkt levels in seven replicate blots as in (A) presented as pAkt/Akt after normalizing to the loading control, actin. **(C)** Ebselen increases insulin-induced glucose uptake in L6-GLUT4 myotubes. Serum-starved myotubes were pretreated with ebselen (25 µM, 20-24 h) and stimulated with insulin (100 nM, 15 min) followed by the glucose uptake assay.** (D)** SHIP2 overexpression abrogates the effect of ebselen to induce glucose uptake. L6-GLUT4 myotubes overexpressing SHIP2 by lentiviral infection were treated and glucose uptake performed as described in (C). Graph D legends are the same as in graph E. **(E)** SHIP2 overexpression abrogates the effect of ebselen to reduce ROS production. L6 myotubes transiently overexpressing SHIP2 were treated with 10 µM ebselen or corresponding volume of DMSO as a control for 20 h and ROS production was detected using DCFH-DA fluorescent probe. Hoechst 33342 was used for normalization. **(F)** Representative immunoblots show that SHIP2 overexpression partially abrogates the effect of ebselen to increase the expression levels of catalase and SOD1. L6 myotubes transiently overexpressing SHIP2 were treated as described in (E). Cell lysates were subjected to immunoblot analysis with anti-SHIP2, anti-catalase, anti-SOD1 and anti-tubulin or anti-actin IgGs. **(G)** Quantification of SHIP2, catalase and SOD1 expression levels in three replicate blots as in (F) normalized to the loading controls, tubulin or actin. Data are presented as means ± SD of three-five independent experiments. Student's *t* test and one- or two-way ANOVA for multiple comparisons. *p ˂ 0.05, **p ˂ 0.01, ***p ˂ 0.001.

To understand the molecular mechanisms by which ebselen enhances insulin sensitivity, we analysed whether ebselen enhances the phosphorylation of Akt, a key enzyme that regulates the activity of the insulin signaling pathway. Treatment of cultured myotubes with ebselen dose-dependently enhanced the insulin-induced phosphorylation of Akt indicative of its activation (Figure 4A, B). To test whether ebselen enhances the insulin-induced phosphorylation of Akt by inhibiting SHIP2, we knocked down SHIP2 in myoblasts leading to an average 70% decrease in the SHIP2 expression level (Supplementary Figure S1F, G). Ebselen and knockdown of SHIP2 increased insulin-induced phosphorylation of Akt by approximately 55% and 33%, respectively (Supplementary Figure S1F, G). In SHIP2 knockdown cells treated with ebselen, Akt phosphorylation was increased by approximately 41% (Supplementary Figure S1F, G), indicating that ebselen fails to potentiate Akt phosphorylation upon SHIP2 depletion. We further found that insulin increases glucose uptake in myotubes by approximately 17%, and ebselen together with insulin further potentiates the effect leading to 35% increase in glucose uptake (Figure 4C). To confirm that ebselen increases glucose uptake by inhibiting SHIP2, we overexpressed SHIP2 in myotubes leading to an average 60% increase in the SHIP2 expression level (Supplementary Figure S1H, J) and approximately 20% decrease in insulin-stimulated glucose uptake (Figure 4D). Ebselen increased glucose uptake by 18% in empty vector-transfected myotubes, while SHIP2 overexpression prevented ebselen-induced increase in the insulin-stimulated glucose uptake (Figure 4D). Taken together, these findings indicate that ebselen increases Akt activity and enhances glucose uptake by reducing SHIP2 activity.

### Ebselen reduces ROS production by inhibiting SHIP2 activity and increases the expression of antioxidant catalase in myotubes

The antioxidant properties of ebselen are attributed to its known radical scavenging activity 19. To investigate whether ebselen decreases ROS production by inhibiting SHIP2 in myotubes, we performed SHIP2 overexpression experiments. SHIP2 overexpression in myotubes increased ROS production by 15% compared to the empty vector-transfected myotubes (Figure 4E). Ebselen decreased ROS levels by 16% in empty vector-transfected myotubes and prevented SHIP2 overexpression-induced increase in ROS in SHIP2 overexpressing myotubes (Figure 4E). In line with decreased ROS levels, we observed that ebselen increases the expression level of catalase and has a tendency to increase SOD1 in myotubes (Figure 4F, G). Overexpression of SHIP2 alone had no significant effect on the expression levels of catalase or SOD1, however, SHIP2 overexpression showed a tendency to decrease the ability of ebselen to increase the expression levels of the antioxidants (Figure 4F, G). Altogether, these results indicate that ebselen reduces the oxidative stress caused by increased expression of SHIP2.

### Ebselen protects liver tissue from lipid peroxidation and inflammation in the db/db mice

**Figure 5:**
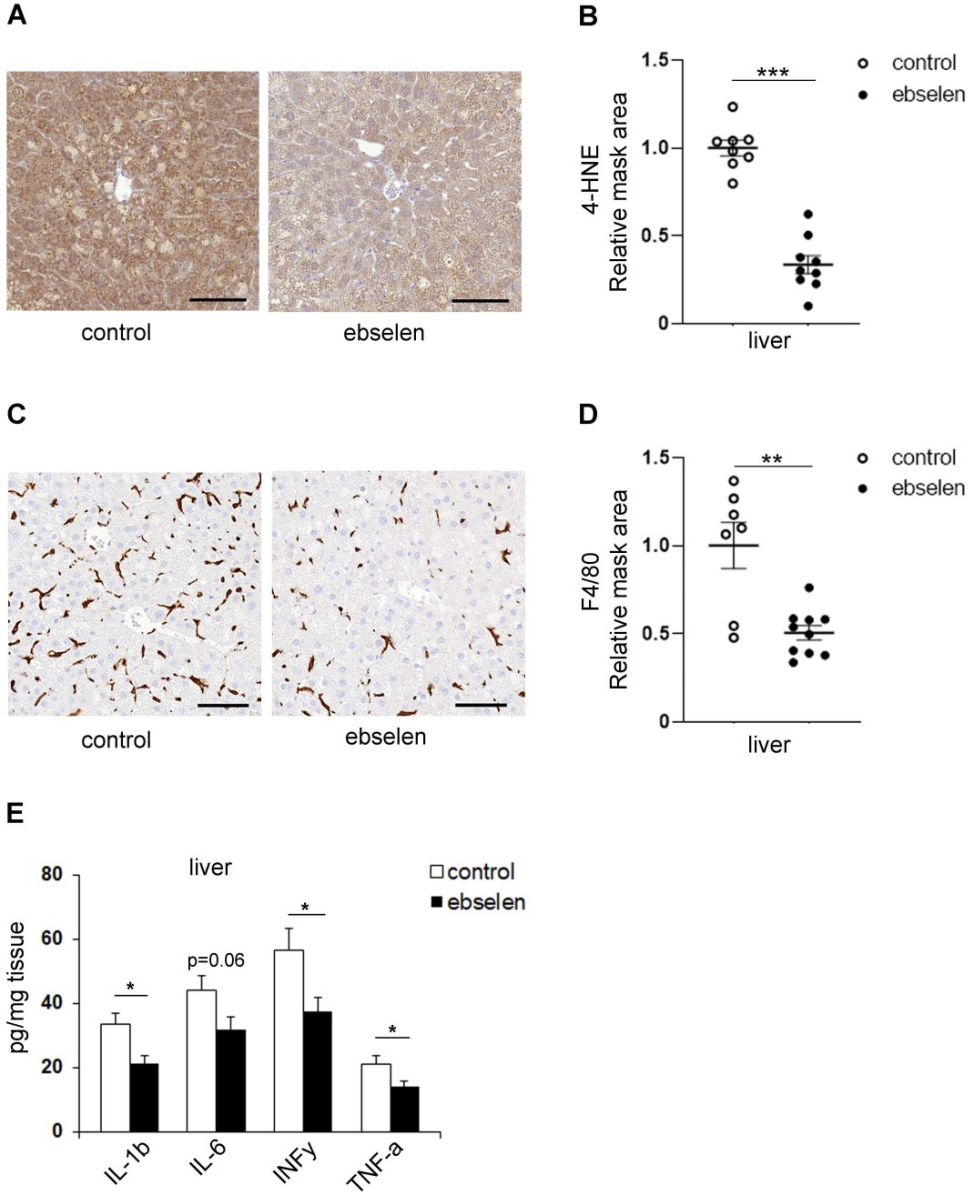
** Ebselen attenuates lipid peroxidation and inflammation in the liver of the db/db mice. (A, C)** Representative images of immunohistochemical stainings of liver sections for lipid peroxidation marker 4-HNE (A) and total macrophage marker F4/80 (C). Paraffin sections of liver samples were processed for immunohistochemical staining and labeled with anti-4-HNE and anti-F4/80 IgGs. Scale bar: 100 µm. **(B, D)** Ebselen decreases the expression levels of 4-HNE (B) and F4/80 (D) in the liver of the db/db mice. Ten randomly chosen liver cross section areas were selected from each mouse for quantification of the 4-HNE (A) and F4/80 (C) positive areas. Quantification was performed with HistoQuant program. **(E)** Ebselen decreases the levels of IL-1β, INFγ and TNFα and shows a tendency to decrease the level of IL-6 (p=0.06) in the liver of the db/db mice. Cytokine levels were measured from liver tissue lysates by an ELISA-based assay. control n=9; ebselen n=10. Data are presented as means ± SEM. Student's *t* test. *p ˂ 0.05, **p ˂ 0.01, ***p ˂ 0.001.

As the PI3K/Akt signaling pathway is a central regulator of hepatic glucose and lipid metabolism that are closely interrelated with inflammatory signaling within liver 30, 31, we analyzed whether SHIP2 inhibition by ebselen affects these pathways in the db/db mice. However, ebselen had no effect on the mRNA levels of the key gluconeogenesis genes, phosphoenolpyruvate carboxykinase (PCK1) and glucose-6-phosphatase (G6Pase) (Supplementary Figure S4A). Ebselen had also no effect on the mRNA levels of transcription factor sterol regulatory element binding protein 1 (SREBP1), a master regulator of lipogenesis (Supplementary Figure S4A). Also, the protein expression levels of fatty acid synthase (FAS) and phosphorylated acetyl-CoA carboxylase (pACC), enzymes involved in fatty acid synthesis, were unaltered after ebselen treatment (Supplementary Figure S3C, D). In line with this, lipid accumulation in the liver of the ebselen-treated db/db mice was not decreased based on the Oil Red O staining (Supplementary Figure S4B, C). Notably, ebselen decreased the expression level of 4-HNE, an oxidative stress marker for lipid peroxidation (Figure 5A, B). Ebselen did not increase the expression levels of catalase and SOD1 (Supplementary Figure S3C, D), or decrease the expression levels of 3-NT, 8-OHdG and fibronectin (p=0.08), markers for nitro-oxidative stress, oxidative DNA damage and fibrosis, respectively, in the liver of the db/db mice (Supplementary Figure S4D-J). Remarkably, ebselen decreased the expression level of F4/80, a total macrophage marker (Figure 5C, D). In line with this finding, the levels of cytokines IL-1β, INFγ and TNFα were decreased and IL-6 tended to decrease (p=0.06) in ebselen-treated liver tissue (Figure 5E) despite that ebselen had no effect on the mRNA expression levels of IL-1α and TNFα (p=0.08) (Supplementary Figure S4A). These data collectively indicate that ebselen protects the liver tissue from lipid peroxidation and inflammation in the db/db mice.

## Discussion

Previously, we performed structure-based virtual screening to identify new SHIP2 inhibitors 17 with better drug-like properties than previously characterized SHIP2 inhibitors 32. Interestingly, we identified glutathione peroxidase mimetic drug, ebselen, that has been examined in clinical trials for managing various diseases, such as cardiovascular diseases, stroke, bipolar disorder and cancer (see clinicaltrials.gov website for more information), and most recently identified 33 and registered for a clinical trial study as a new drug candidate against SARS-CoV-2 coronavirus. In addition to its antioxidant and anti-inflammatory properties, the metabolic role of ebselen in hyperglycemic conditions proposed a new therapeutic application for this compound to treat diabetes 19. Several studies revealed that ebselen lowers hyperglycemia and activates the insulin signaling pathway 21-23, but the exact molecular mechanism remained open. Here, we show that lipid phosphatase SHIP2 is a direct molecular target of ebselen *via* which ebselen improves insulin sensitivity and reduces oxidative stress. We also observed that ebselen reduces inflammation in diabetic liver.

In this study we demonstrate that ebselen reduces potently the enzymatic activity of SHIP2 in cultured myotubes and hepatoma cells, while the known SHIP2 inhibitors, metformin and AS1949490, inhibit SHIP2 only in myotubes 17. We also found that ebselen inhibits SHIP2 in the skeletal muscle and liver of the db/db mice, whereas metformin does not inhibit SHIP2 in the liver of the db/db mice 17 as it targets mitochondrial glycerophosphate dehydrogenase 34. Furthermore, the effect of ebselen and metformin to inhibit SHIP2 in the SHIP2-Tg mice varied depending on the tissue. The distinct outcome of ebselen and metformin in our *in vitro* and* in vivo* studies could be explained by cell and tissue-specific differences and the efficacy of ebselen and metformin. In line with our previous data on metformin 17, decreased SHIP2 activity after ebselen treatment was not due to reduced expression of SHIP2. Thus, our *in vitro* and *in vivo* data indicate that ebselen acts on SHIP2 by reducing its activity without influencing its expression level.

We observed that ebselen slightly improves insulin sensitivity in the db/db mice. This is in line with previous studies with SHIP2 inhibitors metformin 17 and AS1949490 26 in the db/db mice, and ebselen in the Zucker diabetic fatty rats 21 and ischemic gerbils 23, all showing that SHIP2 inhibitors improve insulin sensitivity. Furthermore, ebselen was more effective than metformin in overcoming the impaired glucose metabolism in the SHIP2-Tg mice. These data support the potential of SHIP2 as a target to improve glucose metabolism and the role of ebselen in the treatment of diabetes-related disorders. In line with previous literature in various animal models 21, 23, 35, 36, we observed no difference in body weight in the db/db and SHIP2-Tg mice after ebselen treatment. There was also no difference in fasting blood glucose levels or the expression of the rate-limiting enzymes of gluconeogenesis, although, studies show that ebselen decreases blood glucose levels in Zucker diabetic fatty rats 21 and lowers PCK1 expression in an animal model of ischemic stroke 23. In addition, previous studies in diabetic mice show that liver-specific inhibition of SHIP2 ameliorates hepatic insulin resistance by suppressing gluconeogenesis genes 9, 12. The difference could be due to different animal models and the doses of ebselen used, although emerging studies propose that pathways independent of gluconeogenesis genes can also suppress insulin-mediated hepatic glucose production 37 and gluconeogenesis 38.

Notably, we found that ebselen moderately increases insulin-stimulated glucose uptake in cultured myotubes, supported by earlier data on rat skeletal muscle fibers and cultured adipocytes 21, 22. Here, we show that this occurs by reducing SHIP2 activity, as overexpression of SHIP2 decreased the ability of ebselen to induce insulin-stimulated glucose uptake. Consistent with studies on known SHIP2 inhibitors 26, 39, 40, we also observed that ebselen increased insulin-induced Akt phosphorylation in myotubes, suggesting a role for ebselen in the activation of the PI3K/Akt signaling pathway. This is further strengthened by our data showing the direct binding of ebselen to SHIP2. However, based on the literature showing that ebselen inhibits protein kinase C 41 and inositol monophosphatase 42, we cannot rule out the possibility that ebselen targets also other enzymes involved in the insulin signaling pathway. SHIP2 hydrolyzes PI(3,4,5)P3 to PI(3,4)P2 and both phosphoinositides can activate Akt 43, 44. In addition, other lipid phosphatases modulate the levels of PI(3,4,5)P3 and PI(3,4)P2 5, 45, making it challenging to define the effect of SHIP2 inhibitor treatment on the levels of PI(3,4,5)P3 or other phosphoinositides. In line with this, no difference was observed in the PI(3,4)P2 levels in HEK cells depleted of SHIP2 46 or in mouse embryonic fibroblasts generated from mice deficient of SHIP2 47. Nevertheless, the data described above, together with the observation that ebselen inhibits SHIP2 *in vitro* and *in vivo*, suggest that ebselen improves insulin sensitivity and facilitates glucose uptake by reducing SHIP2 activity.

We found that ebselen decreases serum cholesterol and tends to decrease LDL levels in the db/db mice. Our data showing that ebselen had no effect on liver steatosis or lipogenesis suggest that ebselen has no effect on the lipid synthesis pathway. Similarly, no effect or a trend of ebselen to decrease serum lipid parameters have been observed in diabetic ApoE/GPx1 double knockout mice, a model combining hyperlipidemia and hyperglycemia with increased oxidative stress 36, 48. However, based on our findings and studies showing that serum lipid parameters are improved in the SHIP2 knockout 11 and catalytically inactive SHIP2 49 mouse models, the observed minor effect of ebselen on blood lipid profile could be due to SHIP2 inhibition as overexpression of an inactive form of SHIP2 ameliorates high-glucose-induced de-novo lipogenesis and VLDL (very LDL) production in HepG2 cells 15.

Our data revealing that ebselen decreases the levels of 4-HNE in the liver of the db/db mice are in line with previous literature on various tissues 21, 35, 48, 50, supporting the role of ebselen as a potent inhibitor of lipid peroxidation. Since the other oxidative and fibrosis markers tended to decrease in the liver by ebselen treatment, it would be interesting to test whether a higher dose of ebselen would provide better protection. Nevertheless, we observed that ebselen also upregulated the expression levels of catalase in myotubes and SOD1 in the skeletal muscle of the db/db mice, suggesting that this may be an additional mechanism whereby ebselen reduces oxidative stress. This is interesting as muscle, contrary to liver, typically contains lower levels of first-line defense antioxidant enzymes 51, 52. Similar effect of ebselen has been observed on catalase expression in the diabetic ApoE knockout mice 53 and on SOD activity in a rat model of myocardial ischemia/reperfusion injury 54, a condition that also induces oxidative stress and inflammation in tissues 55, 56. It has been shown that the main antioxidant action of ebselen is to specifically reduce phospholipid and cholesterol hydroperoxides 57, which is crucial in protecting cells from oxidative damage. Indeed, here we found that ebselen decreases ROS production in myotubes cultivated under hyperglycemic conditions and that SHIP2 overexpression abrogates this, indicating that increased expression of SHIP2 contributes to increased ROS levels in myotubes. This is in line with previous studies in hepatocytes showing that catalytically inactive form of SHIP2 protects from palmitate or high glucose -induced excessive ROS production 15, 16. Furthermore, inhibition of the catalytic activity of SHIP2 with AS1949490 in CD2AP-deficient glomerular epithelial cells ameliorates ROS production 25. Collectively, these data suggest that ebselen, in addition to acting as an antioxidant, protects the tissues of the db/db mice from oxidative damage by reducing SHIP2 activity.

Our data demonstrating that ebselen decreased pro-inflammatory cytokines in the liver of the db/db mice are in line with previous studies indicating the role of ebselen in the suppression of liver injury 58, 59. Consistent with these findings, we also observed that ebselen attenuates F4/80-positive macrophage infiltration in the liver of the db/db mice. Studies show that SHIP2 negatively regulates FcγR-mediated phagocytosis in murine macrophages 60 and macrophage colony-stimulating factor-induced Akt activation is required for the survival of various macrophage cell lines 61, while overexpression of phosphatase-defective mutant SHIP2 restores TNFα-induced impaired Akt activity in adipocytes 14. The literature thus supports the function of SHIP2 in the immune system, and our data above propose a mechanism *via* which ebselen acts as an anti-inflammatory agent. However, due to the versatile chemical structure of ebselen, it interacts with a large number of enzymes 41, 42, 62-65 that contribute to its antioxidant and anti-inflammatory properties 19. Indeed, we found that ebselen, in contrast to metformin 17 and AS1949490 26, also inhibits recombinant SHIP1 phosphatase domain, which shares 64% sequence identity with the phosphatase domain of SHIP2. Thus, the anti-inflammatory properties of ebselen observed in the liver of the db/db mice could be attributed to its potential to inhibit SHIP1, which is expressed predominantly, but not exclusively, by immune and hematopoietic cells, and testis 66. This hypothesis is supported by a similar study showing that treatment of diet-induced obese mice with a small-molecule inhibitor of SHIP1, K118, improves blood glucose tolerance and insulin sensitivity, and reverses diet-associated obesity by attenuating inflammation in the visceral adipose tissue 67. Interestingly, the authors also speculate that weight loss in obese mice could be attributed to the inhibition of SHIP2 with K118 67, proposing that both ebselen and K118 are dual-target inhibitors. Taken together, our data suggest that ebselen attenuates inflammation by reducing SHIP2 and SHIP1 activity, consequently leading to improved insulin sensitivity in insulin-sensitive tissues.

Normal regulation of tissue inflammation, ROS generation and PI3K/Akt signaling pathway are required for the maintenance of cell homeostasis and survival 2, 31. Hyperglycemia-induced oxidative stress due to increased ROS production and decreased levels of antioxidants, and inflammation provoked by release of pro-inflammatory cytokines are key factors associated with metabolic diseases and T2D 3. This highlights the need for developing effective drugs to ameliorate insulin resistance as well as oxidative stress and inflammation, and which can be used either as a monotherapy or as part of a combination approach. We propose that ebselen is one of such promising drugs.

Collectively, we show here that ebselen binds directly to the phosphatase domain of SHIP2 and reduces its activity *in vitro* and *in vivo*. This consequently activates the Akt signaling pathway and enhances glucose uptake in myotubes, and increases insulin sensitivity in mice *in vivo*. Ebselen also decreases the production of ROS in myotubes, at least in part, by inhibiting SHIP2 and activates antioxidant enzymes in myotubes and skeletal muscle of the db/db mice. In addition, ebselen reduces lipid peroxidation and inflammation in the liver of the db/db mice. Thus, our findings unravel a novel molecular mechanism by which ebselen ameliorates insulin resistance and protects against oxidative stress and chronic inflammation and highlights the potential of SHIP2 as a drug target to treat metabolic disorders.

## Supplementary Material

Supplementary figures and tables.

## Abbreviations

ACC: acetyl-CoA carboxylase
CETSA: cellular thermal shift assay
FAS: fatty acid synthase
G6Pase: glucose-6-phosphatase
HDL: high-density lipoprotein
3-NT: 3-nitrotyrosine
4-HNE: 4-hydroxynonenal
8-OHdG: 8-hydroxy-2'-deoxyguanosine
IL-1α and β: interleukin 1 alpha and beta
IL-6: interleukin 6
IFNγ: interferon gamma
LDL: low-density lipoprotein
NEFA: non-esterified fatty acid
PI(3,4)P2: phosphatidylinositol (3,4)-bisphosphate
PI(3,4,5)P3: phosphatidylinositol (3,4,5)-trisphosphate
PCK1: phosphoenolpyruvate carboxykinase 1
SHIP1: Src homology 2 domain-containing inositol-5-phosphatase 1
SHIP2: Src homology 2 domain-containing inositol-5-phosphatase 2
SOD1: superoxide dismutase 1
SREBP1: sterol regulatory element binding protein 1
T2D: type 2 diabetes
TNFα: tumor necrosis factor alpha
