# Supplementary material for: Ebselen enhances insulin sensitivity and decreases oxidative stress by inhibiting SHIP2 and protects from inflammation in diabetic mice
Source: Int J Biol Sci. 2022 Feb 14;18(5):1852–64. doi: 10.7150/ijbs.66314 (PMC8935241; doi:10.7150/ijbs.66314)
Supplement: Supplementary file 1 — Supplementary figures and tables. [file ijbsv18p1852s1.pdf]

Supplementary Material

Polianskyte-Prause *et al.*: Ebselen enhances insulin sensitivity and decreases oxidative stress by inhibiting SHIP2 and protects from inflammation in diabetic mice

Supplementary Table S1. Antibodies used in the study.

antibody	species	provider
SHIP2	goat<br>mouse	Santa Cruz Biotechnology (Dallas, TX, USA)
8-OHdG	mouse	
phospho Akt (S473)	rabbit	Cell Signalling Technology (Danvers, MA, USA)
phospho ACC (Ser79)	rabbit	
F4/80	rabbit	
Pan Akt	mouse	R&D Systems (Minneapolis, MN, USA)
tubulin	mouse	MilliporeSigma (Burlington, MA, USA)
actin	rabbit	Abcam (Cambridge, UK)
ACC	rabbit	
Catalase	rabbit	
FAS	rabbit	
4-HNE	rabbit	
3-NT	mouse	
SOD1	rabbit	
Fibronectin	rabbit	
IRDye 680	rabbit	LI-COR Biosciences (Lincoln, NE, USA)
IRDye 800	mouse	
Alexa Fluor 680	goat	Invitrogen (Carlsbad, CE, USA)

Supplementary Table S2. Primers used in the study.

gene	primer sequence
PCK1	forward-5´- CGTGGCCGAGACTAGCGATC-3`
	reverse 5´- ATAATGGGGCACTGGCTGGC-3´
G6Pase	forward 5´- AACGCCCGTATTGGTGGGTC-3´
	reverse 5´- GGCATGGCCAGAGGGACTTC-3´
TNF-α	forward 5´- CTTCTGTCTACTGAACTTCGGG-3´
	reverse 5´- CAGGCTTGTCACTCGAATTTTG-3´
IL1- α	forward 5´- TGCCACCTTTTGAGAGTGATGAGA-3´
	reverse 5´- TGCCTGCCTGAAGCTCTTGT-3´
SREBP1α	forward 5´- CATGGACGAGCTGGCCTTCG-3´
	reverse 5´- GGAAGTCACTGTCTTGGTTGTTGA-3´
18S rRNA	forward 5´- GAGGGACAAGTGGCGTTCAG-3´
	reverse 5´ - ATCACGAATGGGGTTCAACG-3´

Supplementary Table S3. Body weight and blood glucose of SHIP2-Tg mice after 12 days of ebselen or metformin treatment. Ebs, ebselen; Met, metformin.

	SHIP2-Tg	SHIP2-Tg +Ebs	SHIP2-Tg	SHIP2-Tg +Met
**Body weight (g)**	24.6±0.6	24.4±0.5	30±0.5	30±0.6
**Blood glucose (mmol/L)**	7.2±0.3	7.5±0.3	6.6±0.2	6.3±0.3

Data are presented as means ± SEM (control n=8-9; ebselen n=10; metformin n=8).

Supplementary Table S4. Body weight, food consumption and ebselen intake of the db/db mice during the treatment period. Ebs, ebselen.

	db/db	db/db+ Ebs	db/db	db/db+ Ebs	db/db	db/db+ Ebs	db/db	db/db+ Ebs
**Age (weeks)**	7	7	10	10	14	14	16	16
**Duration of treatment (weeks)**	3 days	3 days	4	4	8	8	10	10
**Body weight (g)**	34±0.9	34.2±0.9	46.5±0.8	46.6±1.0	54.5±1.1	54.5±1.0	55.7±1.0	56±1.1
**Food consumption (g/day)**	6.6±0.2	6.1±0.2	6.9±0.1	6.5±0.2	6.4±0.1	5.8±0.1**	6.3±0.2	6.1±0.1
**Ebselen intake/ weight (mg/kg)**		13.4		10.5		8		8.2

Data are presented as means ± SEM (control n=9; ebselen n=10) (**$p < 0.01$).

Supplementary Table S5. Blood glucose and serum lipid profile of the db/db mice after 10 weeks of ebselen treatment.

	db/db	db/db+Ebs
**Blood glucose (mmol/L)**	30.5±0.7	30.6±0.9
**Triglycerides (mmol/L)**	4.5±0.4	4.4±0.4
**Cholesterol (mmol/L)**	4.2±0.1	3.7±0.2*
**LDL (mmol/L)**	0.5±0.05	0.4±0.02[a]
**HDL (mmol/L)**	1.9±0.05	1.8±0.09
**NEFA (mmol/L)**	1±0.05	1±0.06

Data are presented as means ± SEM (control n=9; ebselen n=10). (*$p < 0.05$, [a] $p = 0.08$).

Supplementary Figures

Figure S1

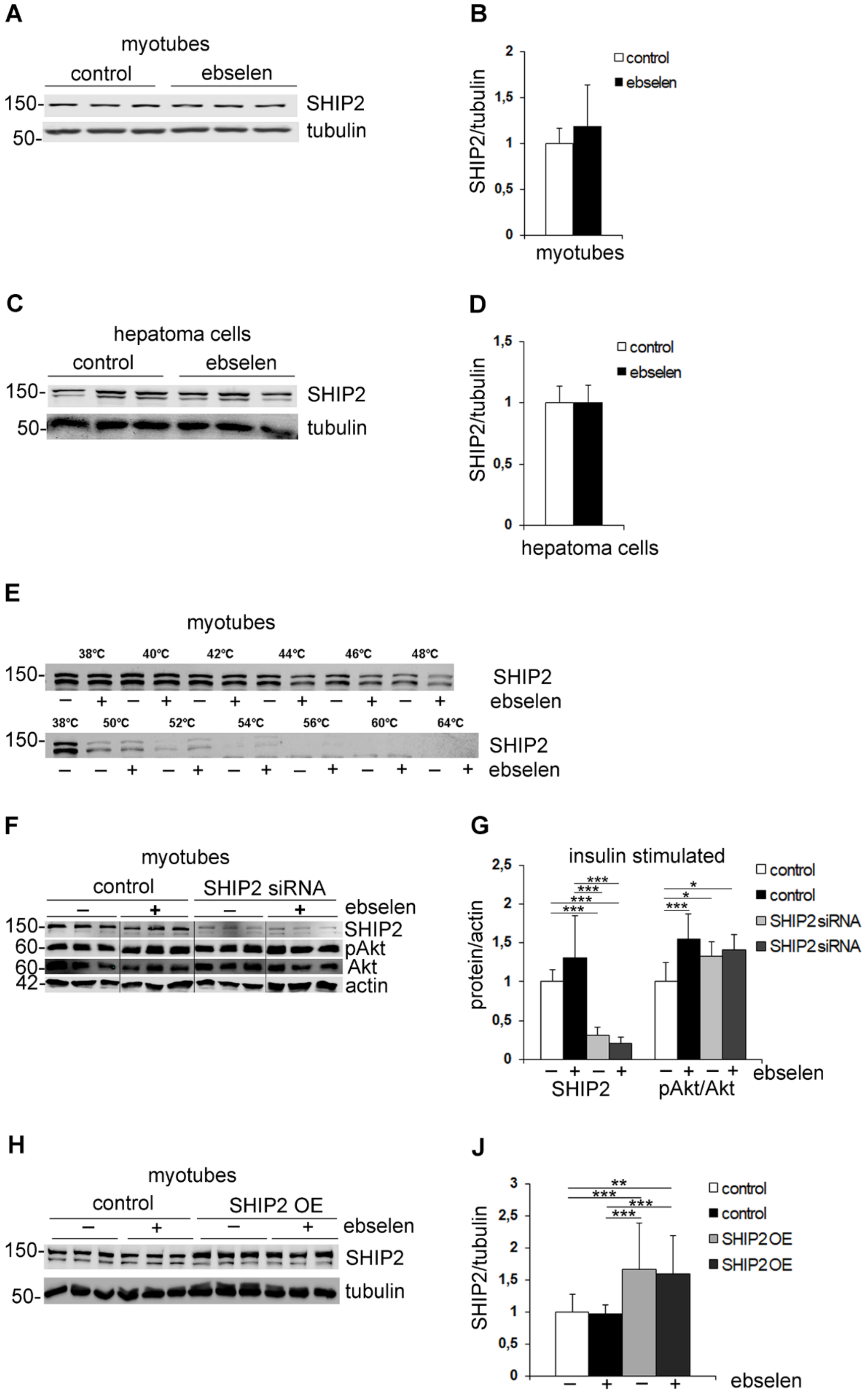

Supplementary Figure S1. Ebselen does not affect the expression level of SHIP2 in myotubes and hepatoma cells or in myotubes overexpressing SHIP2, induces a shift in the melting temperature of SHIP2 and enhances insulin signaling in myotubes. (A, C) Representative immunoblots show that ebselen has no effect on the expression level of SHIP2 in L6 myotubes (A) and hepatoma cells (C). Cells were treated with 10 µM ebselen and a corresponding volume of DMSO as a control for 20-24 h and lysates were subjected to immunoblot analysis with anti-SHIP2 and anti-tubulin IgGs. (B, D) Quantification of SHIP2 levels in (A, C) normalized to loading control, tubulin. (E) Representative immunoblot for SHIP2 in myotube lysates treated with ebselen used for CETSA quantifications. Myotube cell lysates were treated with 50 µM ebselen or corresponding volume of DMSO as a control for 15 min followed by heat treatment. Soluble fractions of the cell lysates were subjected to immunoblot analysis with anti-SHIP2 IgG. (F) Representative immunoblot for SHIP2, pAkt and Akt in L6 cells transfected with *INPPL1* siRNA or control siRNA. Ebselen and siRNA-mediated SHIP2 silencing increase insulin-induced Akt phosphorylation (pAkt) in L6 cells. In L6 myotubes silenced for SHIP2, ebselen fails to potentiate Akt phosphorylation. Serum-starved cells (20 h) were pretreated for 15 min with 10 µM ebselen or corresponding volume of DMSO as a control followed by stimulation with 10 nM insulin for an additional 15 min. Cell lysates were subjected to immunoblot analysis with anti-SHIP2, anti-Akt, anti-pAkt (Ser 437) and anti-actin IgGs. (G) Quantification of SHIP2 levels in (F) normalized to loading control actin reveals decreased SHIP2 levels in siRNA transfected cells. Quantification of pAkt levels in (F) presented as pAkt/Akt after normalizing to loading control, actin. (H) Representative immunoblot for SHIP2 in L6-GLUT4 myotubes overexpressing SHIP2 or empty vector (control) by lentiviral infection. Cells were treated with 25 µM ebselen and a corresponding volume of DMSO as a control for 20-24 h and lysates were subjected to immunoblot analysis with anti-SHIP2 and anti-tubulin IgGs. (J) Quantification of SHIP2 expression level in (H) normalized to loading control, tubulin, reveals increased SHIP2 level in SHIP2-overexpressing cells. Mouse anti-SHIP2 IgG was used for immunoblots in (A) and goat anti-SHIP2 IgG for immunoblots in (C, E, F, H). Data are presented as means ± SD of three-four independent experiments. Student's *t* test and two-way ANOVA for multiple comparisons. $^{*}p < 0.05$, $^{**}p < 0.01$, $^{***}p < 0.001$.

Figure S2

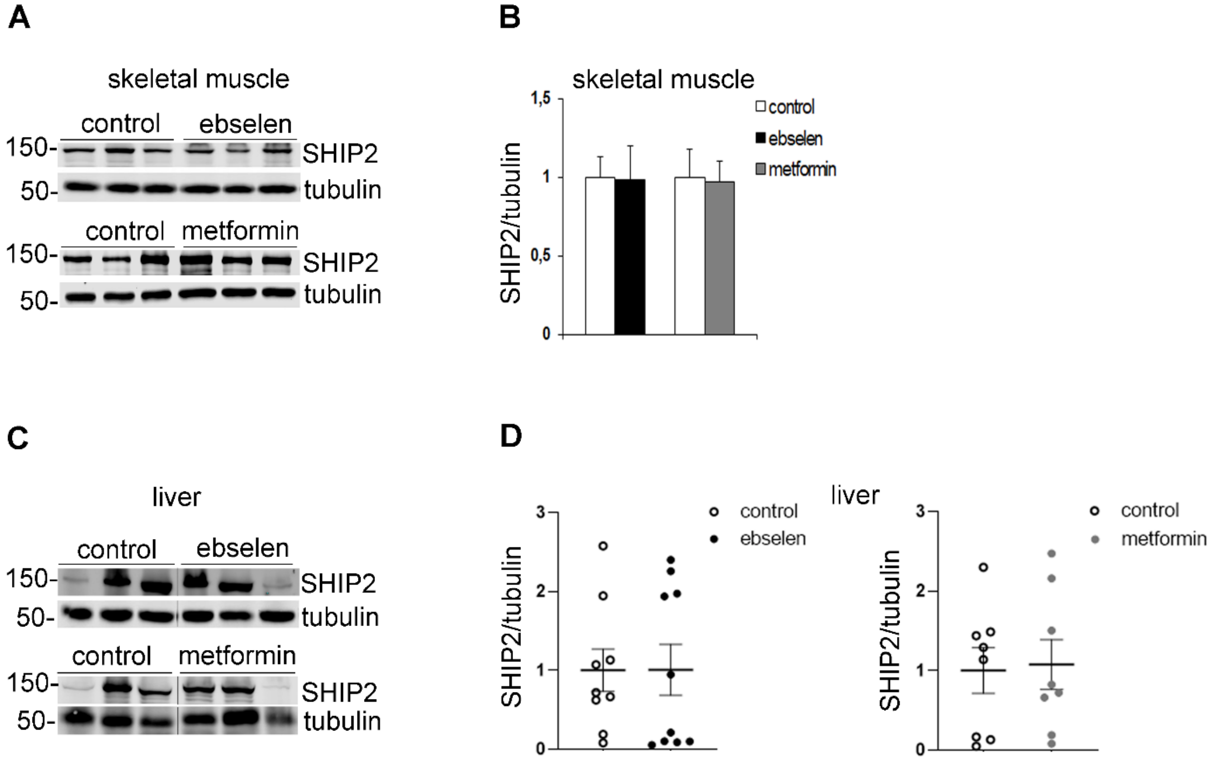

Supplementary Figure S2. Ebselen does not affect the expression level of SHIP2 in the tissues of the SHIP2-Tg mice. (A, C) Representative immunoblots show that ebselen and metformin have no effect on the expression level of SHIP2 in the skeletal muscle (A) and liver (C) of the SHIP2-Tg mice. Tissue lysates from ebselen- or metformin-treated and corresponding control SHIP2-Tg mice were subjected to immunoblot analysis with anti-SHIP2 and anti-tubulin IgGs. Vertical lines in the immunoblots for SHIP2 and tubulin in (C) visualize merging of different parts of the same membranes. (B, D) Quantification of SHIP2 expression levels in (A, C) normalized to loading control, tubulin. Data are presented as means ± SEM control n=5-9; ebselen n=10; metformin n=8.

Figure S3

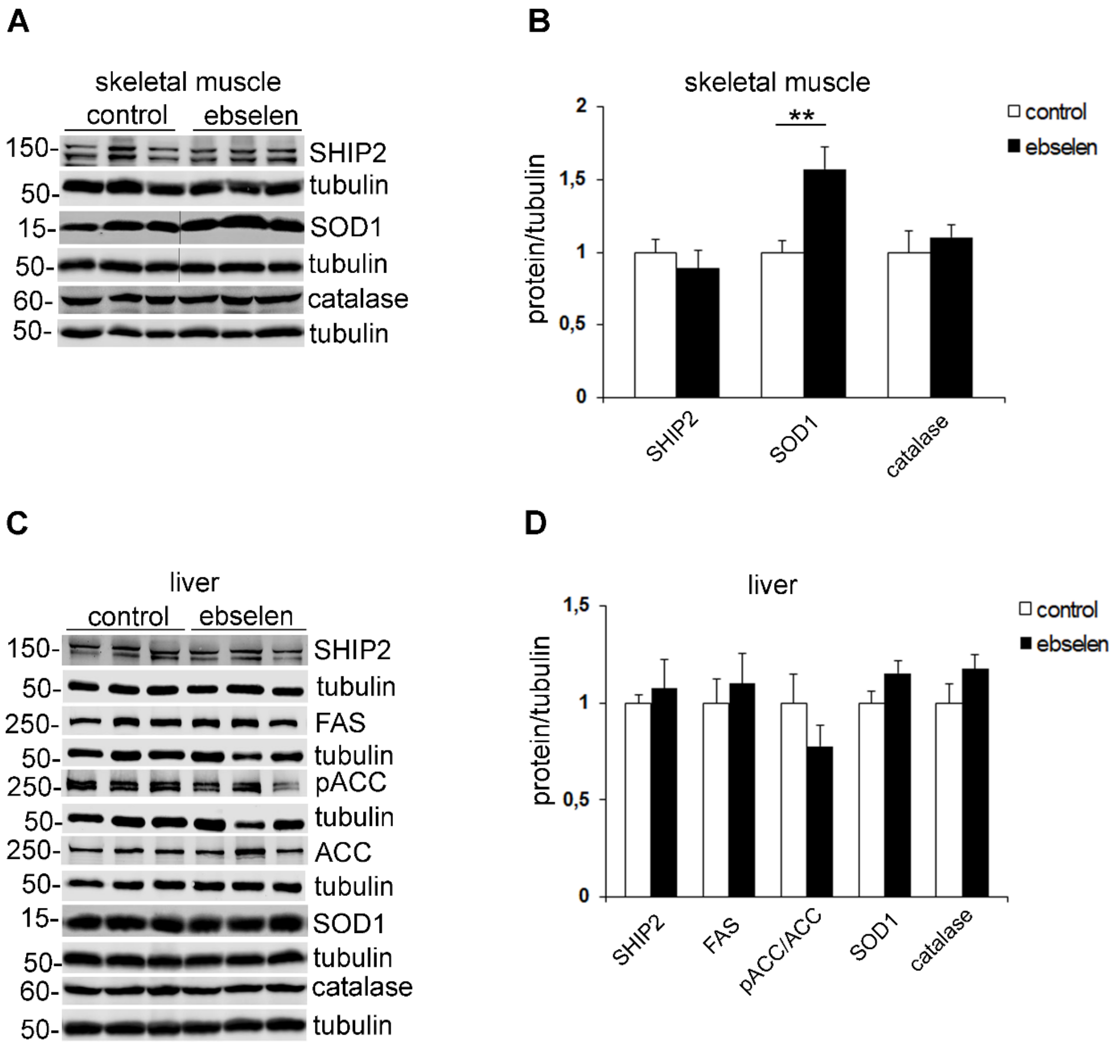

Supplementary Figure S3. Ebselen does not affect the expression level of SHIP2 in the tissues of the db/db mice or the expression levels of FAS, ACC and catalase in the skeletal muscle and liver of the db/db mice, and increases the expression level of SOD1 only in the skeletal muscle of the db/db mice. (A) Representative immunoblots show that ebselen does not affect the expression levels of SHIP2 and catalase, and increases the expression level of SOD1 in the skeletal muscle of the db/db mice. Tissue lysates from ebselen-treated and corresponding control db/db mice were subjected to immunoblot analysis with anti-SHIP2, anti-catalase, anti-SOD1 and anti-tubulin IgGs. The vertical lines in the immunoblots for SOD1 and tubulin visualize merging of different parts of the same membranes (B) Quantification of SHIP2, catalase and SOD1 expression levels in (A) normalized to loading control, tubulin. (C) Representative immunoblots show ebselen has no effect on the expression levels of SHIP2, FAS, catalase and SOD1 and the phosphorylation of ACC (pACC) in the liver of the db/db mice. Immunoblotting was performed as described above. (D) Quantification of SHIP2, FAS, catalase and SOD1 expression levels in (C) normalized to loafing control, tubulin. Quantification of pACC level in (C) presented as pACC/ACC after normalizing to loading control tubulin. Data are presented as means ± SEM control n=8; ebselen n=9. Student's *t* test. **p < 0.01.

Figure S4

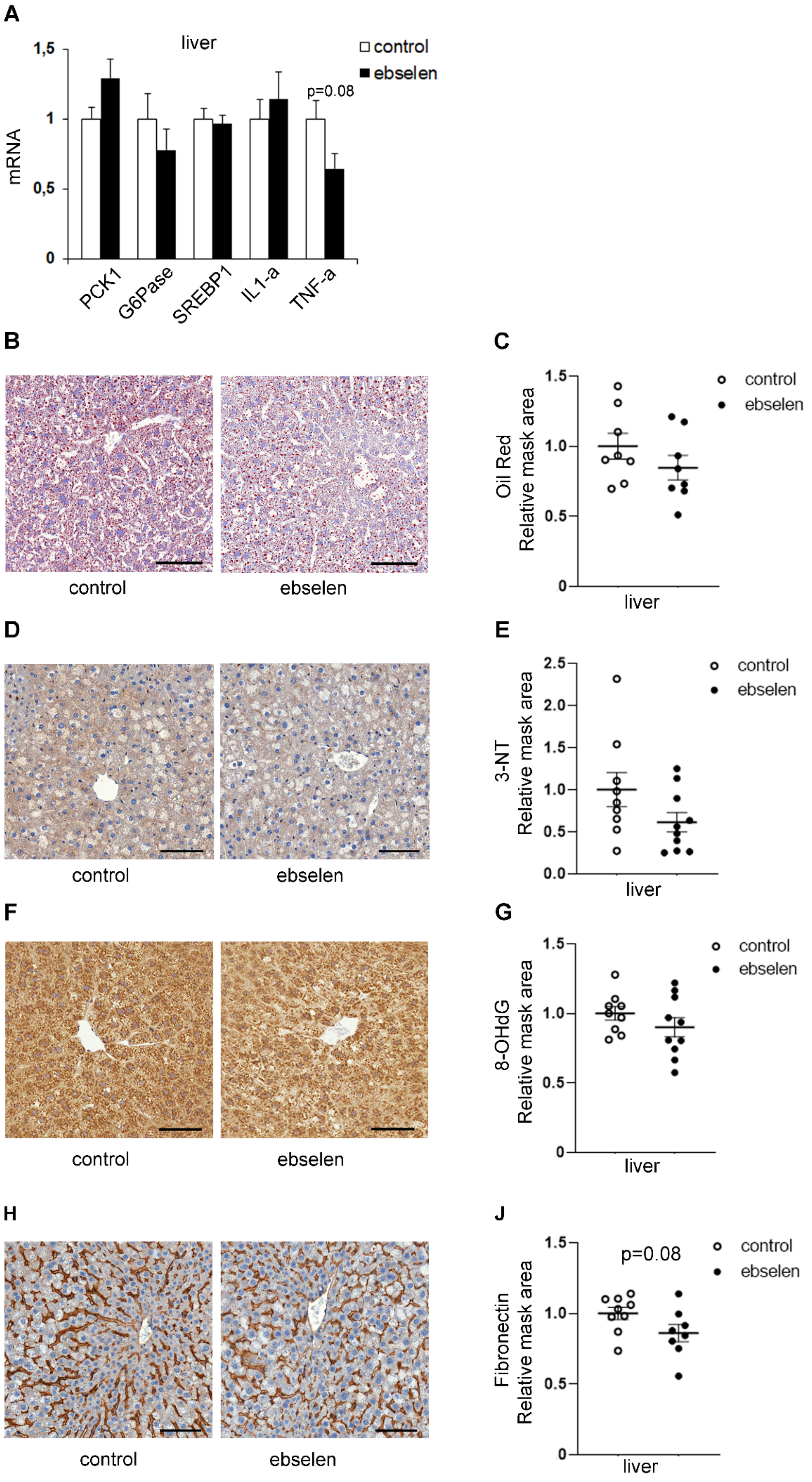
A
liver
control
ebselen
p=0.08
mRNA
1,5
1
0,5
0
PCK1
G6Pase
SREBP1
IL1-a
TNF-a
B
control
ebselen
C
Oil Red
Relative mask area
liver
control
ebselen
D
control
ebselen
E
3-NT
Relative mask area
liver
F
control
ebselen
G
8-OHdG
Relative mask area
liver
H
control
ebselen
J
p=0.08
Fibronectin
Relative mask area
liver

Supplementary Figure S4. Ebselen has no effect on the gene expression levels of gluconeogenesis, lipogenesis and inflammation markers, steatosis or the expression levels of nitro-oxidative stress, oxidative DNA damage and fibrosis markers in the liver of the db/db mice. (A) Ebselen does not affect the expression of PCK1, G6Pase, SREBP1, IL-1α and TNFα (p=0.08) genes. The expression levels of the rate limiting gluconeogenesis genes PCK1 and G6Pase, lipogenesis gene SREBP1 and inflammation genes IL-1α and TNFα in the liver were analyzed by quantitative RT-PCR. S18 rRNA was used as an internal control. The bars show the mean expression in arbitrary units. n=6 per group. (B, D, F, H) Ebselen does not decrease lipid accumulation (B) or the expression levels of peroxynitrite-mediated oxidative damage marker 3-NT (D), oxidative DNA damage marker 8-OHdG (F) and fibrosis marker fibronectin (H) in the liver of the db/db mice. Frozen or paraffin sections of liver samples were processed for immunohistochemical staining with Oil Red O or labeled with anti-3-NT, anti-8-OHdG and anti-fibronectin IgGs, respectively. (C, E, G, J) Quantification of Oil Red O (B), 3-NT (D), 8-OHdG (F) and fibronectin (H) positive area in the liver of the db/db mice was performed with HistoQuant program. Ten randomly chosen liver cross section areas were analyzed from each mouse. Data are presented as means ± SEM. Scale bar: 100 μm.
